# De Omnibus Rebus Et Quibusdam Aliis

**Published:** 1854-02

**Authors:** 


### ART. III.—"*De omnibus rebus et quibusdam aliis.*" By Rusticus.

Messrs. Editors:—One might have deemed, from the tone of my last epistle, that I considered a resurrected theory worthless—that because an idea had once figured on the current of opinion, and then disappeared, that it was *de facto* unworthy of a subsequent consideration. From any such interpretation of the drippings of my brain, excuse me. I have an honest veneration for the labors of that medical period when Sir Christopher Wren and others were engaged in vivisection and experiment. It is true that these old worthies proved, sometimes, too much. Their ignorance of absorbent action generated some queer blunders.

Thus, in a paragraph upon Hydrops Pectoris, by Natt Fairfax, about A. D. 1670, he argues of pleuritic effusion, that "'T is probable that the Liquor proceeded from the falling down of the *Chyle* from the *Axillars.*" In a paper on the same subject, by Mr. Samuel Doudy, he suggests the operation of paracentesis thoracis in pleuritic effusion, in these words: "Perhaps it may not be impracticable to use the *Paracenthesis* in the like case, when the disease is certainly known, and without it death is most likely to ensue."

The light of auscultation has given to us, at last, the power of "certainly knowing" the disease; and Dr. Bowditch is, by argument and reports of cases, urging it upon the profession, and now, after a lapse of nearly two hundred years from the first proposal of this operation, it is likely to become an approved means of cure in those cases supposed by Doudy, where "death is most likely to ensue."

It was after these publications of Fairfax and Doudy, viz., on "Jun. 21, 1683," that Dr. Will. Musgrave (the endorser of the Haematokinetic doctrines of that day,) injected water into the thorax of a dog. The water was absorbed, and the dog recovered; whence our accurate experimenter derives the following bit of theory:

"Perhaps, as nature has furnished us Vessels to bring off that humour which is thrown into the *Ventricles* of the *Brain*, and by tarrying there would prove fatal to us, so likewise there may be some *Ductus* yet unknown (to me at least) which belonging to the Thorax, may convey off thence what Liquor arises (either from the Condensation of Vapours, or from the Rupture of *Lymphaticks*, or any other Way,) in the cavity, mediately or immediately, into the Blood: Certainly these Experiments, as also the Hystories of *Empyemad's*, and *Dropsies of the Breast*, mention'd by Physicians as cur'd by

Evacuations by Urine, do, in some measure, argue the Probability of such a Passage."

It would seem that Musgrave was here upon the verge of the discovery of the absorbent system. The lymphatics were already known, but it was thought that they were employed, not in the collection of surplusage in the system, but in the distribution of nutrient matter. M. Louy de Bill's discourses theranent.

" * * * most of the Juice of the *Milky Vessels* is discharged between the *Tunicles* of the *Veins*, *Arteries*, *Lymphaticks*, *Membranes*, and the *Vessels* in the *Mesentary*, to be conveyed into all the parts of the Body, both Internal and External. * * * * The remaining and least Part of the *Juice* of the *Milky Vessels* is transmitted through the *Ductus Thoracicus* by the *Jugular Vein* into the Blood."

The subject of dropsies was to the old physicians a very interesting one, and the distinction between ovarian and abdominal dropsies was early made.

Dr. Robert Houstoun reports an operation for ovarian dropsy, made in August, 1701, which may well rival any of those since performed. If boldness and daring are the highest qualities of the surgeon, then does this Glasgow chirurgeon deserve high mention for his "achievement." *Seipse dicit.*

" * * * with an Imposthume Lancet I laid open about an inch; but finding nothing issue, I enlarged it to two inches, and even then nothing came forth but a little thin yellowish Serum; so I ventured to lay it open two Inches more. I was not a little startled, after so large an aperture, to find it stopp'd only by a glutinous Substance. All my Difficulty was to remove it; I tried my Probe, I endeavored with my Fingers, but all was in vain; it was so slippery that it eluded every Touch, and the strongest Hold I could take."

"I wanted, in this Place, every thing necessary, but bethought myself of a very odd Instrument, yet as good as the best, because it answered the End proposed. I took a strong Firr-Splinter, wrapped some loose Lint about the End of it, and thrust it into the Wound, and by turning and winding it, I drew out above two Yards in Length of a substance thicker than any Gelly; or rather like Glue that is fresh made and hung out to dry; the breadth of it was above ten Inches. This was followed by nine full Quarts of such matter as I have met with in Steatomatous and Atheromatous Tumours, etc."

This woman recovered. It is, so far as I know, the earliest successful operation for ovarian dropsy by removal of the organ. He states further on, that he removed "several large Pieces of Membrane which seemed to be part of the distended Ovary."

I hardly know why I am thus detailing to you these histories of long ago. They teach their lesson however, but each reader must search it for himself; for I am in no sermonizing mood, and am only wandering at my own sweet will among the curiosities of medical literature. If your readers are not interested in all this, they can turn to the abundance of wiser articles, among which my own jottings down are embalmed in your pages.

Among the most curious of medical edicts extant, is one which my pen hesitates to copy, lest it break its limbs upon the ancient Gaelic words. It has, however, its bearing upon a disputed point in medical history, viz: the introduction of syphilis into Europe. The antiquity of the disease is here fully vindicated, and the old opinion, that it was brought from America by the companions of Columbus, and propagated by them at the siege of Naples, in 1495, is disproved by the fact that the *Grandgor* was prevalent in Edinboro in 1497. Communication, in those days, was so slow between Scotland and southern Europe, that is impossible that syphilis could have traveled that distance, and invaded so large a share of the population as it evidently had at that time.

"22 Septr. 1497.

"It is our Soverance Lords Will and the Command of the Lordis of his Counsale send to the Provost and Bailies within this bur't that this Proclamation followand be put till execution for the eschewing of the greit appearand danger of the Infection of his Lieges fra this contagious sickness callit the *Grandgor* and the greit uther Skayth that may occur to his Lieges and Inhabitans within this bur't; that is to say we charge straitly and commands be the authority above written, that all manner of personis being within the freedom of this bur't quilks are infectit or hes been infectit uncurit with this said contagious plage callit the *Grandgor*, devoid, red and pass furt of this Town and compeir upon the sandis of *Leith* at ten hours before none (10, A.M.) and thair sall they have and fynd Botis (*boats*, gentle reader!) reddie in the havin ordanit to them be the Officeris of this bur't reddely furneist with victuals to have thame to the *Inche*,* and thair to remane quhill God proviyd for thair Health:"

Furthermore, this proclamation orders all curers of this disease to go with their patients, and if they cure any one suffering with it anywheres "in Edinboro town," "ilk ane of them salle be brynt on the cheik with the marking Irne that they may be kennit in tym to cum, and thairafter gif any of tham remainis that thai sall be banist but favors."

---

* An Island in the frith of Edinboro, opposite Leith.

This humane, and to the medical profession, highly honorable proclamation, is from James the IVth, as appears in the records of the Town Council of Edinboro.

An older document, preserved in the Bishops' Court of Winchester, dates still farther back. It was written about A. D, 1430, and is conclusive that syphilis was well known more than sixty years before the siege of Naples. In the easy morals of those days, the Church derived a goodly income from the licensing of stews, and the sums received in fines from them. Some earlier regulations of the Lord Bishops, dating some time in 1162, gave directions as to all the sanatary precautions to be observed in stews. The document first alluded to as dating in 1430, commences: "Here begyne the Ordinances, Rules, and Cusstumes as well for the Salvation of Mannes Lif, as for to aschieve many Myschiefs and Inconvenients, that dayley be lik there to fall owte, to be rightfully kept, and due Execution of them to be dou unto any Personne within the same.

"*Item.*—That no Stew-holder keep noo Woman wythin his hous that hath any Sycknesse of BRENNING, but that she be putte owte upon the peyne of makeit a fyne unto the Lord of a hundred Shyllyings."

I had intended, Messrs. Editors, to write this essay on the subject of Transfusion of Blood, its history, and its claims to confidence as an important and neglected therapeutic means. Some other time I may do so; but now, in making the necessary inquiries as to its early history, I have stumbled upon so many quaint specimens of early medical literature, that I have sketched them for you, without regard to the patience of your readers. I was puzzled for an appropriate heading to these pages, and have, at last, scored out the original, and placed another there, which assuredly covers the "whole entirety" of my scribblement.

—— × Roads, Jan. 1, 1854.

